# Application of organoids in otolaryngology: head and neck surgery

**DOI:** 10.1007/s00405-023-08348-4

**Published:** 2023-12-13

**Authors:** Hai Zhu, Siyuan Qu, Yongqin Deng, Mengdan Gong, Yizhen Xiang, Yaoshu Teng, Dong Ye

**Affiliations:** 1 Department of Otorhinolaryngology-Head and Neck Surgery, The Affiliated Lihuili Hospital, Ningbo University, Ningbo, 315040 Zhejiang China; 2 Department of Otorhinolaryngology, Affiliated Hangzhou First People’s Hospital, Zhejiang University School of Medicine, Hangzhou, 310006 Zhejiang China

**Keywords:** Organoids, 3D tissue model, Tumor prediction model, Stem cells, Otolaryngology—head and neck surgery

## Abstract

**Purpose:**

The purpose of this review is to systematically summarize the application of organoids in the field of otolaryngology and head and neck surgery. It aims to shed light on the current advancements and future potential of organoid technology in these areas, particularly in addressing challenges like hearing loss, cancer research, and organ regeneration.

**Methods:**

Review of current literature regrading organoids in the field of otolaryngology and head and neck surgery.

**Results:**

The review highlights several advancements in the field. In otology, the development of organoid replacement therapies offers new avenues for treating hearing loss. In nasal science, the creation of specific organoid models aids in studying nasopharyngeal carcinoma and respiratory viruses. In head and neck surgery, innovative approaches for squamous cell carcinoma prediction and thyroid regeneration using organoids have been developed.

**Conclusion:**

Organoid research in otolaryngology—head and neck surgery is still at an early stage. This review underscores the potential of this technology in advancing our understanding and treatment of various conditions, predicting a transformative impact on future medical practices in these fields.

## Introduction

### Definition and characteristics of organoids

Organoids, as miniature tissue and organ analogs, have three characteristics, including self-assembly, various cell types, and similar to the internal organs in structure and function to a great extent [1]. Organoids are cultured in vitro with 3D technology, in which multicellular masses can highly simulate the physiological and pathological structure and tumor cell heterogeneity of tissues or organs in vivo [2, 3].

**Fig. 1:**
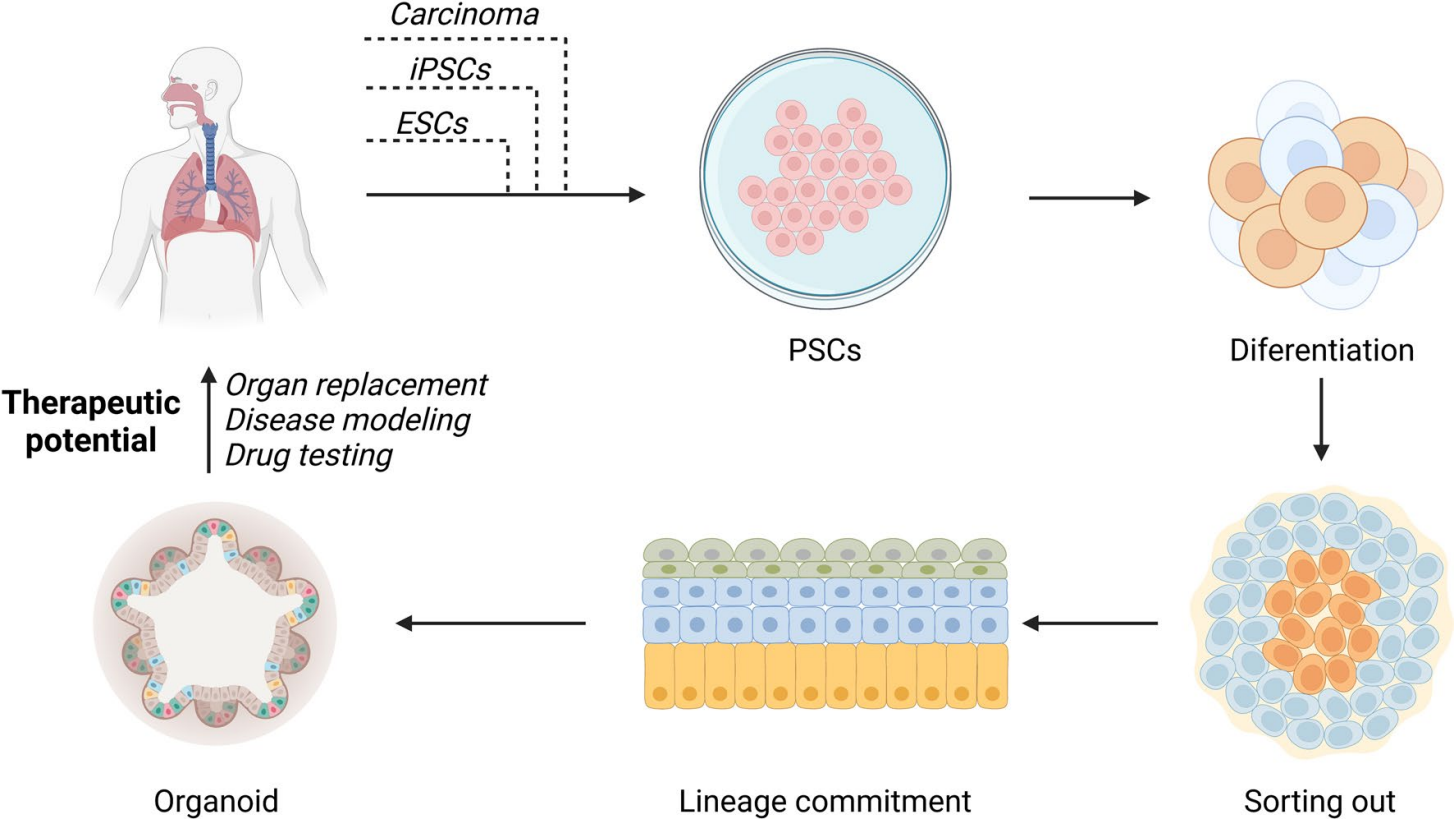
The acquisition, construction and application of organoids

As organoids of various organs and tissues, such as intestines, stomach, liver and kidney [4–7], have been successfully cultured in vitro, the huge potential of organoid technology has been continuously developed. Organoid technology provides in vitro conditions for understanding the mechanism of development of tissues and organs, disease development and precision medicine. In addition, it can be used for drug toxicity detection, efficacy evaluation and new drug screening. However, there is still a lack of systematic synthesis of studies on organoid technology in otolaryngology, head and neck surgery. Therefore, this paper summarized the latest research results of organoids in otolaryngology—head and neck surgery and discussed the future development of organoid technology. The acquisition, construction and application of organoids are shown in Fig. 1.

### Establishment of organoid model

**Fig. 2:**
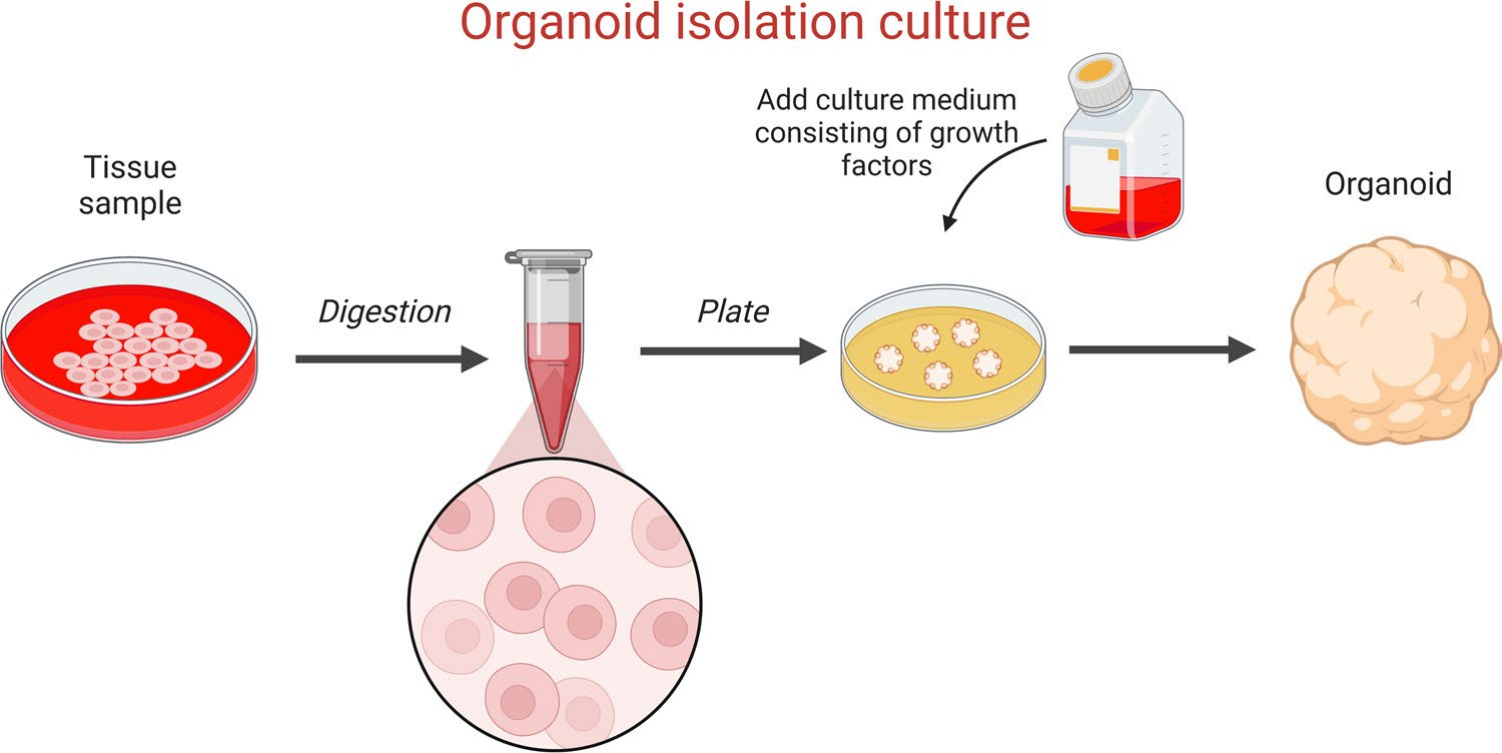
Organoid construction process

Organoids are mainly cultured from stem cells, including pluripotent stem cells, adult stem cells and tumor stem cells. Presently, most organoid modeling methods require stem cells, matrigel and cytokine-rich medium, which can be established in an average of 10–14 days. The organoid construction process is shown in Fig. 2.

#### Preparation of tissue

Organoids can be produced from solid materials, such as surgical specimens, puncture biopsy specimens and nasal brush specimens, or liquids like urine, ascites and bronchoalveolar lavage fluid [8–11]. For solid specimens, the first step is to remove the non-epithelial tissue (such as muscle or fat) as much as possible, then use a scalpel to cut it into 1–3-mm small pieces, digest the tissue with enzymes and separate the epithelial cells.

#### Inoculation of cells

The isolated cells or small cell masses are inoculated into 3D extracellular matrix (ECM) hydrogels, such as basement membrane extract (BME), Matrigel or Geltrex, which can be used as artificial lamina propria [12].

#### Organoid culture

After inoculation, cells are supplemented with a medium consisting of a mixture of growth factors that trigger regenerative or damage responses in epithelial tissue stem cells. The key components include: (i) activators of Wnt signaling, such as Wnt ligand and LGR5 ligand R-spondin (RSPO) [13–15]; (ii) tyrosine receptor kinase ligands, such as epidermal growth factor (EGF), capable of promoting epithelial cell value addition [16, 17]; and (iii) inhibitors of the transforming growth factor-β/osteogenic protein signaling pathway, such as Noggin [18], which induces epithelial differentiation.

## Current research

### Otology research

More than 6% of the world's population suffers from hearing loss and balance impairment [19]. Both sensory systems are located in the inner ear and can be affected by aging, genetic mutations, infections, noise exposure and ototoxic drugs. Hearing loss is irreversible, and there are currently no medications that specifically target sensory recovery. As a 3D multicellular system that simulates the inner ear in vitro, inner ear organoids are promising new tools to realize cell replacement therapy and understand the inner ear nerve cells [20, 21].

#### Culture of inner ear organoids from pluripotent stem cells

Unlike other organoids, the inner ear is difficult to biopsy and grow for a long time [22], so patient-sourced tissue cannot be used, and using fetal-sourced tissue has ethical issues. Therefore, human pluripotent stem cells (hPSCs) may be a potential source of tissue cells for experiments.

hPSCs differentiate into ear progenitor cells and more mature inner ear cells by mimicking embryonic and fetal development [11, 23, 24]. In embryos, the development of the inner ear requires the participation of multiple cell types from multiple cell lineages, including inner ear epithelial cells, neuron cells and glial cells from the ectoderm, and periauricular mesenchymal cells from the mesoderm [25]. The challenge is synthesizing these multicell lines into an inner ear organoid in vitro, which is a long-term bioengineering challenge.

As an extremely complex organ, the inner ear is formed by integrating many signal pathways across space and time. These signals come from the inner and surrounding tissues of the epithelial cells, which make the cochlear progenitor cells differentiate into cochlear and vestibular cells. Most of our knowledge of these mechanisms comes from animal models, and very little has been done on human fetal inner ear tissue [26]. To some extent, the self-assembly of inner ear epithelial cells and neuronal complexes can be stimulated by using recombinant proteins and small molecules to simulate signals in hPSC 3D culture [20]. However, this approach is difficult to control, and the resulting organoids are of irregular shape and size and contain an unpredictable mix of sensory and non-sensory cells. In future studies, more sophisticated 3D bioprinting-based or microfluid-based approaches may be needed to build spatially controlled cell structures that can be affected by signal gradients to create an inner ear organoid chip.

Recent studies have found that the use of microfluidics or microwell systems to enable hPSCs to form embryonic-like, renal or intestinal structures can guide studies to induce inner ear formation [9, 27–29].

#### Inner ear organoids simulate hereditary deafness

It is estimated that 430 million people worldwide suffer from moderate to severe hearing loss [30, 31]. The most permanent hearing loss is of the sensorineural type (SNHL), and the causes include aging [32], infection [33, 34], noise [35, 36], ototoxic drug [37, 38], traumatic tympanic membrane rupture [39] and single gene mutation.

Although the etiology of SNHL is largely established, its underlying pathophysiological mechanisms have not been fully elucidated at the cellular and molecular levels. Therefore, the use of inner ear organoids to model hereditary deafness is a very valuable application.

There are generally two approaches to in vitro modeling of hereditary deafness. The first involves using CRISPR-Cas9 to introduce deafness-related mutations into wild embryonic stem cell (ESC) lines [40, 41], guided editing [42] or other precision genome editing techniques. The second is to obtain somatic cells from patients with inherited deafness and induce them to be transformed into induced pluripotent stem cells (iPSCs) [43, 44] and then gradually induce iPSCs or CrisPR-Cas9-edited ESCs to differentiate into inner ear-like tissues. The use of iPSCs clearly has greater therapeutic potential than ESCs, as the use of IPSC-derived donor cells in the treatment of the inner ear can avoid rejection.

Researchers have modeled two types of autosomal recessive non-syndromic deafness, DFNB2 and DFNB3, using a 2D culture system based on hiPSC [45, 46]. However, the organoid-based 3D culture system can easily perform single-cell RNA sequencing (RNA-SEQ) on inner ear-like tissues. Tang et al. investigated human hearing loss caused by mutations in the gene encoding type II transmembrane protease 3 (TMPRSS3) using inner ear organoid and scRNA-seq, revealing a potential role for calcium homeostasis and extracellular matrix maintenance in TMPRSS3-associated deafness [47].

### Rhinology research

#### Nasopharyngeal carcinoma organoid disease model

The nasopharyngeal carcinoma (NPC) is a tailor-made malignant tumor with a high geographical prevalence in the top and lateral walls of the nasopharynx and is closely correlated with Epstein–Barr virus infection. For patients with NPC at the beginning of stage I, II, III and IV, the 5-year survival rate is more than 70% after comprehensive treatment with radiotherapy and chemotherapy [48], but 25% of NPC patients fail due to local recurrence and distant metastasis [49]. Studies have shown that tumor stem cells are closely related to the occurrence, development, recurrence and metastasis of cancer. They can not only self-renew but also have the resistance effect to traditional chemoradiotherapy. Therefore, it is valuable to screen out chemotherapeutic drugs that NPC stem cells sensitive to.

Tumor organoids are the tumor tissues of patients cultured in vitro in 3D, and tumor cells with the potential of stem cells will converge and grow spherical, thus forming organoids with the ability of self-renewal and self-organization. Compared with traditional cell lines and xenotransplantation, tumor organoids have the advantage of individualization. In addition, the tissue needs are less, and the culture cycle is shorter. It is helpful to screen chemotherapy drugs or targeted drugs accurately and efficiently [50]. However, it does not have a vascular and immune environment, so it still has limitation when used to screen immunotherapy drugs.

Patient-derived xenografts (PDXs) have been used in NPC studies, but the low success rate and high cost of PDXs limit their large-scale application [51, 52]. NPC tissues are usually obtained by endoscopic biopsy, and their small tissue size and poor cell activity pose major challenges in Nasopharyngeal carcinoma organoid (NPCO) culture. Wang et al. established a patient-derived organoid model, and an optimized medium can significantly increase the success rate of NPCO culture, preserve parental tumor heterogeneity and reproduce its pathophysiological features [53].

However, there are still many challenges to generating NPCOs, including the overgrowth of fibroblasts. There are many infiltrating lymphocytes in NPC tissues [54]. It has been reported that T and B lymphocytes can secrete cytokines to control the growth of fibroblasts [55, 56]. However, after several passages, the immune cells in NPCOs would stop growing and die, resulting in decreased concentrations of TGF-β and TNF-α, which could not inhibit fibroblast growth [57]. Further studies are needed to understand how to inhibit the growth of fibroblasts to prolong the passage of NPCOs.

#### Nasal organoid respiratory virus model

Respiratory organoids are often used as an in vitro airway model to study the pathogenesis of respiratory viruses and test therapeutic methods [58]. However, respiratory organoids technology needs to use invasive methods to obtain patient samples. Rajan et al. reported a non-invasive technique using human nasal organoids (HNOs) as an alternative to tissue-derived organoids [59].

HNOs were cultured in an air–liquid interface (ALI), and the infection of two major human respiratory viruses, including respiratory syncytial virus (RSV) and novel coronavirus (SARS-CoV-2), was evaluated, reproducing the complex host-virus interaction. SARS-CoV-2 causes severe damage to cilia and epithelial cells [60–62], no interferon-I response and little mucus secretion. In contrast, RSV causes mucous hypersecretion and severe interferon-I response with ciliary body damage.

Chiu et al. also reported the establishment of a nasal organoid model to study the infection of SARS-CoV-2. They further reproduced the higher infectability and replication adaptability of the Omicron variant showing its pathogenesis, such as the destruction of ciliary cells and tight connections, to promote the spread and development of the virus [63].

The nasal organoid respiratory virus model, which simulates upper respiratory tract infections and effectively reconstructs human nasal epithelium in a stable culture plate, provides microbiologists with a powerful and convenient tool to study the pathogenesis and test treatments for the current epidemic of SARS-CoV-2 and its emerging variants.

### Pharyngology, head and neck surgery research

#### Head and neck squamous cell carcinoma prediction model

Head and neck malignancies are the seventh most common tumor types worldwide, among which more than 90% are [64]. The emergence of therapies, including targeted therapies and immunotherapies, is increasing the need to test treatment options in personalized settings. Currently, new treatments for HNSCC are mainly tested at the population level, meaning that within a group of patients, multiple subgroups with different efficacy and side effects are included. This makes it difficult to predict how well a therapy will work for an individual patient. Consequently, new therapies are often only tested as palliative treatments in patients with advanced HNSCC.

HNSCC organoids fill the gap in personalized prediction of treatment outcomes. In a specific culture environment, tumor tissue can be grown into organoid models to test different treatments, predict treatment outcomes, and then treat patients. In addition, it could allow new drugs to be tested before entering clinical practice.

#### Tissue-derived thyroid organoid model

Total thyroidectomy as a treatment for thyroid cancer can cause hypothyroidism and require patients to take thyroid hormones for life. Ogundipe et al. isolated cells from mouse and human thyroid tissue and developed an in vitro 3D culture system [65]. It was demonstrated that both mouse and human thyroid cells could be isolated, expanded in vitro and cultured for a long time. Furthermore, these cells were able to self-renew and differentiate in vitro, suggesting that these cells had the proliferative capacity required for expansion. After transplanting a few cells, these organoids formed fully functioning hormone-producing thyroid follicles in mice with hypothyroidism.

There are two important aspects of thyroid organoid culture that warrant further investigation with this technique. Firstly, organoids can be amplified while maintaining genetic stability [66], proving that they have certain safety as grafts. Secondly, thyroid organoids of patients who have undergone radical surgery for thyroid cancer can be generated from cryo-stored stem cells, which can come from bone marrow or adipose tissue [67].

## Conclusions

The establishment of various organoid models provides new models in vitro for drug research, disease research and organ replacement therapy, with great potential. However, its research in otolaryngology—head and neck surgery is still at an early stage, and its application is relatively limited. Future studies should consider expanding the application scope of this technology, including providing personalized drug treatment for various tumors through tumor organoid technology. Furthermore, by combining organoid technology with 3D printing technology, organoid technology is more closely associated with the regeneration of various organ tissues. It is believed that further development of otolaryngology—head and neck organoid culture systems will have a huge prospect in the future of basic research and translational medicine.

## Data Availability

Not applicable.

## Abbreviations

ECM: Extracellular matrix
BME: Basement membrane extract
RSPO: R-spondin
EGF: Epidermal growth factor
hPSCs: Human pluripotent stem cells
SNHL: Sensorineural type hearing loss
ESC: Embryonic stem cell
iPSCs: Induced pluripotent stem cells
RNA-SEQ: RNA sequencing
TMPRSS3: Transmembrane protease 3
NPC: Nasopharyngeal carcinoma
PDXs: Patient-derived xenografts
NPCO: Nasopharyngeal carcinoma organoid
HNOs: Human nasal organoids
ALI: Air–liquid interface
RSV: Respiratory syncytial virus
SARS-CoV-2: Novel coronavirus
HNSCC: Head and neck squamous cell carcinoma
